# Functional Loss and Mortality in Randomized Clinical Trials for Amyotrophic Lateral Sclerosis: To Combine, or Not to Combine—That is the Estimand

**DOI:** 10.1002/cpt.2533

**Published:** 2022-02-17

**Authors:** Ruben P.A. van Eijk, Kit C.B. Roes, Inez de Greef‐van der Sandt, Leonard H. van den Berg, Ying Lu

**Affiliations:** ^1^ Department of Biomedical Data Science and Center for Innovative Study Design School of Medicine Stanford University Stanford California USA; ^2^ Department of Neurology UMC Utrecht Brain Centre University Medical Centre Utrecht Utrecht The Netherlands; ^3^ Department of Health Evidence Section Biostatistics Radboud Medical Centre Nijmegen The Netherlands; ^4^ Treeway BV Tilburg The Netherlands

## Abstract

Amyotrophic lateral sclerosis is a rapidly progressive disease leading to death in, on average, 3–5 years after first symptom onset. Consequently, there are frequently a non‐negligible number of patients who die during the course of a clinical trial. This introduces bias in end points such as daily functioning, muscle strength, and quality of life. In this paper, we outline how the choice of strategy to handle death affects the interpretation of the trial results. We provide a general overview of the considerations, positioned in the estimand framework, and discuss the possibility that not every strategy provides a clinically relevant answer in each setting. The relevance of a strategy changes as a function of the intended trial duration, hypothesized treatment effect, and population included. It is important to consider this trade‐off at the design stage of a clinical trial, as this will clarify the exact research question that is being answered, and better guide the planning, design, and analysis of the study.

Amyotrophic lateral sclerosis (ALS) is a relatively rare and rapidly progressive disease causing loss of voluntary muscle function and leading to death in, on average, 3–5 years after first symptom onset.^1^, ^2^ A key challenge in clinical trials for ALS is the extensive clinical heterogeneity between patients,^3^, ^4^ where survival time may range from a few months to over 20 years.^5^ Traditionally, increasing the patient’s life expectancy has been the primary objective of randomized clinical trials in ALS. Such studies require, however, relatively long study durations and large sample sizes.^6^, ^7^ In more recent clinical trials, supported by the patient community,^8^ a shift can be observed toward studies with a shorter follow‐up.^9^ In these settings, where study durations commonly range from 6 to 12 months, showing an improvement in overall survival has become impractical.^10^ This has changed the primary focus of ALS clinical trials to intermediate end points such as daily functioning, muscle strength, or quality of life.

Nevertheless, even in these shorter clinical trials, frequently a non‐negligible number of patients die during the course of the study.^11^, ^12^ Although randomization protects the treatment comparison before or at randomization, the occurrence of death after randomization is directly related to the patient’s disease progression^5^, ^13^, ^14^ and may be influenced by the treatment itself. As such, these associations with death could introduce bias in study end points that are based on function. At a patient level, we are often left wondering what would have happened had the patient not died: Would ALS have progressed functionally; would the treatment have protected the patient from further progression; or would death have occurred had the patient been randomized to a different arm.

In the past, there have been extensive discussions about how to handle death in common end points such as the Amyotrophic Lateral Sclerosis Functional Rating Scale–Revised (ALSFRS‐R) or vital capacity.^12^, ^13^ Proposed solutions range from simple imputations to complex algorithms which simultaneously integrate death with the end point of interest. An often overlooked aspect, however, is that the choice of how we address death alters the research question and impacts the clinical interpretation of the study results. Therefore, prior to the discussion about which strategy for handling death may be “best” or “most optimal,” there is a need to define the exact research question that we seek to answer.^15^, ^16^, ^17^

The question to be answered may not always be straightforward.^17^ A patient, for example, may be most interested in how treatment improves their daily functioning during life. The strategy to address death, therefore, must reflect a method that provides the “unbiased” effect of treatment on the patient’s daily functioning during life. In contrast, a physician, payer, or regulator may be much more interested in the totality of the treatment effect. As such, a preferred strategy would be a method that summarizes the total treatment effect on daily functioning and survival, for example, by using a composite end point. Both strategies answer a fundamentally different research question, namely: “What is the effect of treatment on daily functioning *during life*?” *vs*. “What is the effect of treatment on daily functioning *and survival*?” The answer to each of these questions may be different, possibly leading to different conclusions from the same study results. Clarifying the exact scientific objective of a study will not only help guide the discussion about which strategy is required to handle death, but also direct the planning, design, and analysis of clinical trials, and the definition of primary and secondary objectives.

In this paper, therefore, we outline how our choice to handle death in the analysis may change the interpretation of the trial results, illustrated by clinical trials in ALS. We aim to provide a general overview of the main considerations together with a discussion of the most commonly used strategies. We will position these discussions in the estimand framework.^15^, ^16^, ^18^

## THE ESTIMAND FRAMEWORK

The estimand framework, as outlined in the ICH E9(R1) addendum (International Council for Harmonization of Technical Requirements for Pharmaceuticals for Human Use Addendum on Estimands and Sensitivity Analysis in Clinical Trials to The Guideline on Statistical Principles for Clinical Trials, E9(R1)),^19^ aims to help investigators define the exact research question and to align the trial objectives with the study design, including end points and analysis.^20^, ^21^ The estimand, meaning “what needs to be estimated to address the research question,” can be conceptualized as a systematic framework to phrase the different components of the research question. The estimand includes five attributes: (i) the treatment, i.e., a description of the intervention and its comparator, (ii) the population, i.e., the patients to whom the results should apply, (iii) the variable, i.e., the efficacy end point that is required to answer the research question, (iv) the strategy, i.e., a description of how to account for postrandomization events, and (v) the population‐level summary, i.e., the summary statistic that reflects the treatment effect in the efficacy end point.^22^ An explicit term in the estimand framework is postrandomization event or “intercurrent event.” These are events that occur after treatment initiation, and affect either the interpretation or the existence of the measurements associated with the clinical question of interest.^18^, ^23^ Examples include use of concomitant treatments, changes in background treatments, treatment discontinuation, or the occurrence of terminal events, such as death.

The analytical strategy to account for these intercurrent events directly impacts the estimated treatment effect. Thus, based on the research question that we want to answer, we need to consider what strategy best aligns with our objectives. In general, the estimand framework distinguishes five different (example) strategies to address intercurrent events: (i) treatment policy, (ii) hypothetical, (iii) composite, (iv) while on treatment (hereafter referred to as while alive), and (v) principal stratum.^19^ In the treatment policy strategy, one ignores the event and uses the data as is. An example is treatment discontinuation: We would use all available follow‐up data of that patient, irrespective of whether the patient actually underwent treatment or not (i.e., similar to the “intention‐to‐treat” principle). Strictly speaking, we should include the data on the variable of interest after the event. In case of death, however, such a strategy becomes infeasible as the data simply do not exist and we can’t ignore its occurrence.^20^, ^24^, ^25^ Accounting for death using the principal stratum strategy, thereby targeting the patients in whom death does not occur and estimating counterfactual outcomes between treatment arms, is hardly ever used.^26^ In this paper, therefore, we will focus primarily on the following strategies to account for death in ALS clinical trials: (i) the hypothetical strategy, envisaging a hypothetical world where death does not occur, (ii) the while‐alive strategy, summarizing the treatment effect while the patient is alive, and (iii) the composite strategy, making death part of the primary end point of the study.

## THE HYPOTHETICAL STRATEGY

In **Table** **1**, we provide an overview of all randomized controlled clinical trials in ALS published between 2018 and 2021, together with the strategy applied to account for death when evaluating the ALSFRS‐R, the most commonly used efficacy end point in ALS.^9^ In 6 of the 14 studies (43%), a hypothetical estimand was targeted. The treatment effect being estimated is the expected improvement in ALSFRS‐R at the end of the trial, if the patient survives the treatment period. If death rates are low, this estimand has clinical value from a patient perspective, e.g., “If you continue to take the treatment and survive the coming X months, you can expect an efficacy response of Y points in your ALSFRS‐R total score at month X.” In this case, the population‐level summary is the mean difference in ALSFRS‐R total score between treatment and placebo at month X. In order to estimate this effect size, however, all patients must complete the trial. The key challenge for this estimand is, therefore, that the occurrence of death is seen as a missing data problem, and we need to make an assumption for patients who died prior to the end of the study. Importantly, we must assume that the time of death is independent of treatment in order for the estimand to be clinically relevant. In fact, the relevance of this estimand may be the real “hypothetical” part: Can we act hypothetically as if death did not occur and still answer a clinically relevant research question?

**Table 1:** Overview of strategies to handle death in randomized clinical trials in ALS between 2018 and 2021

Author (Year)	Phase	Drug	Sample size	No. deaths (%)	Duration (weeks)	Method	Strategy
Ahmadi (2018)	II	Nanocurcumin	54	5 (9%)	52	Last score	Hypothetical
Aizawa (2021)	II	Perampanel	65	1 (2%)	48	MMRM	Hypothetical
Benatar (2018)	II	Arimoclomol	38	13 (34%)	52	Mixed model	While alive
Berry (2019)	II	Mesenchymal stem cells	48	0 (0%)	24	*—*	*—*
Chen (2020)	II	Tamoxifen	18	2 (11%)	52	MMRM	Hypothetical
Cudkowicz (2021)	III	Levosimendan	496	44 (9%)	48	Joint rank	Composite
de la Rubia (2019)	II	EH301	32	2 (6%)	16	Survivor analysis	Hypothetical
Kaji (2019)	III	Methylcobalamin	373	73 (20%)	182	Worst score	Hypothetical
Ludolph (2018)	III	Rasagiline	252	75 (30%)	78	Linear regression	While alive
Meininger (2017)	II	Ozanezumub	303	14 (5%)	48	Joint rank	Composite
Mora (2019)	III	Masitinib	394	33 (8%)	48	Zero score	Composite
Paganoni (2020)	II	Phenylbutyrate‐Taurursodiol	137	7 (5%)	24	Mixed model	While alive
Statland (2019)	II	Rasagiline	80	9 (11%)	52	Mixed model	While alive
van Es (2020)	II	Penicillin G‐Hydrocortisone	16	6 (38%)	52	Joint model	While alive
Vucic (2021)	II	Dimethyl fumarate	107	1 (1%)	36	Multiple imputation	Hypothetical

Randomized, placebo‐controlled clinical trials, published between January 2018 and November 2021, which included at least 12 weeks of treatment. Crossover studies were excluded.

ALS, amyotrophic lateral sclerosis; MMRM, mixed model for repeated measures.

As can be seen in **Table** **1**, a variety of suggestions has been proposed to impute patient outcomes after death, all leading to potentially different estimates of the treatment effect. In the most simplistic case, we could simply ignore the data of deceased patients and solely use the data from patients who survive until month X (“survivor” or “complete case” analysis). For the population‐level summary to be valid, however, we have to assume that the patients who die are not different from patients who survive, and that outcomes would have been similar at the end of the trial. Apart from the fact that this assumption could be contested in ALS, another disadvantage is that one uses only the data from survivors, which leads to loss of statistical power in an already rare disorder. As an alternative, we could attempt to impute the missing observations at month X for deceased patients, where two common methods include (i) last observation carried forward, or (ii) worst score imputation. In the first strategy, we assume that if the patient hadn’t died during our study, the patient’s condition would not have progressed after their last follow‐up visit. In the second strategy we assume that all deceased patients would have had the worst ALSFRS‐R score observed among surviving patients at month X.

The disadvantage of the above‐mentioned methods is that one either makes a group‐level assumption, e.g., all patients who die receive score Z at month X, or that we make a too simplistic individual assumption in a known progressive and highly heterogenous disorder. As we will discuss next in “the while‐alive strategy,” mixed models for repeated measures (MMRM) partially overcome these limitations. The MMRM assumes that a patient who dies would have had similar outcomes at month X compared with a patient with similar ALSFRS‐R scores up to the time of death, but who survived.^27^

## THE WHILE‐ALIVE STRATEGY

The critical issue with the hypothetical estimand is the fact that it loses its relevance when death rates increase, irrespective of which method is chosen. For example, if only 50% of the patients survive until month X, how relevant is it for a patient to know their expected improvement in ALSFRS‐R at month X if they are unlikely to be alive at that time anyway? Therefore, as an alternative, we could estimate the treatment effect while the patient is alive. This changes the information communicated to the patient. For example: “If you continue to take the treatment while you are alive, you can expect an efficacy response of Y% reduction in your ALSFRS‐R progression rate after X months or until death (whichever occurs first).” In this case, the population‐level summary has changed from a mean difference *at* month X in the hypothetical estimand to a percent reduction in progression rate *after* X months or until death (whichever occurs first). Note that we also switch in the while‐alive strategy to progression rates rather than the actual value of our end point, and the effect size is no longer linked to any particular timepoint. As a consequence, the treatment effect applies to a patient who survives X months, but also to a patient who dies prior to that time.

From **Table** **1** we can observe that the while‐alive estimand was targeted in 36% of the ALS clinical trials. Regression models are a helpful method for estimating the average progression rate in the treatment and placebo arm. The treatment effect, subsequently, can be simply summarized as either an absolute or relative reduction in the disease progression rate compared with placebo. In general, there are currently two main methods for calculating the average progression rate: a two‐stage approach, calculating, for each patient, their individual progression rate and averaging rates across patients (e.g., Ludolph, 2018, **Table** **1**), or a one‐stage approach, defining a random‐slopes model with random effects per patient (e.g., Benatar, 2018, **Table** **1**).

**Figure 1:**
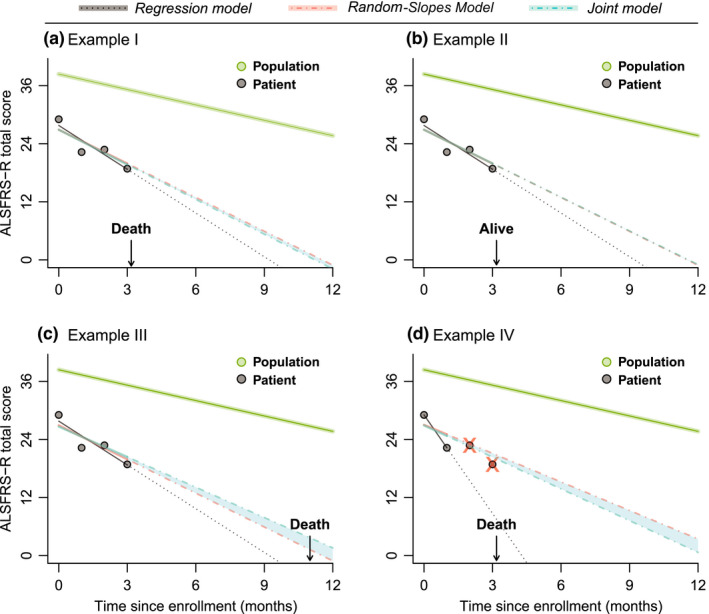
Comparison of different models for the estimated patient trajectory after death. Illustration of hypothetical patient data with varying reasons for and timing of dropout. The *green* line represents the overall population trajectory over time (e.g., the average of all patients in a well‐defined cohort, or patients with ALS allocated to a treatment arm). (**a**) In the first scenario, the patient dies between Month 3 and Month 4. As can be seen, in a random‐slopes model (*red*), the patient’s individual regression line (*black*) is drawn or shrunk toward the population average, based on the amount of information available. (**a**–**c**) A joint model (*blue*) provides a similar estimate compared with the random‐slopes model, but alters the shrinkage factor based on the reason and timing of dropout. The blue shaded area highlights the difference between the random‐slopes and joint model. In scenario (**d**) we illustrate how the model estimates change if the last two observations prior to death are not observed. ALSFRS‐R, Amyotrophic Lateral Sclerosis Functional Rating Scale–Revised.

The difference between the two methods is illustrated in **Figure** **1** for a hypothetical patient. The disadvantage of the two‐stage approach is that when only a few data points are available, this could lead to extreme estimates (e.g., **Figure** **1d**). Moreover, while the patient who dies will provide far less information overall, when estimating the group average rate of decline, a deceased patient and a patient who survives contribute equally. The one‐stage approach using a random‐slopes model improves on this by adjusting, or shrinking, the estimated patient‐specific regression line toward the population mean, based on the amount of information available. The MMRM is similar to the random‐slopes model, with the main difference being an MMRM models time as categorical variable. As a consequence, the MMRM provides the mean difference *at* a certain timepoint or visit, reflecting the hypothetical estimand, whereas a random‐slopes model provides the difference in progression rates *over* a certain time period. Hence, the main benefit of the random‐slopes model is that it provides an effect estimate applicable not only to patients who die prior to a certain timepoint but also to those who survive. In contrast, the MMRM provides an effect estimate applicable only to those patients who survive until a certain timepoint. A disadvantage of the random‐slopes model is, however, that we not only need to make an assumption about the time trajectory (e.g., linear, quadratic, cubic) but also about the treatment effect over time, which is not required for the MMRM.

In **Figure** **1** we can see that the reason why data are missing, or the timing of death occurring do not affect the one‐stage or two‐stage approach. For the model, it simply does not matter whether the patient dies after the last observed data point, or whether they simply withdraw from the study. This may introduce bias,^28^, ^29^ and—also from a clinical perspective—it would be important to consider why the data are missing. This additional information can be incorporated by using a different class of models called shared‐parameter or joint models.^12^, ^13^, ^30^ A joint model is identical to a random‐slopes model, but rather than optimizing model parameters using only the ALSFRS‐R data, it also incorporates information on survival time. As such, model parameters, including the individual trajectory of the patient, are estimated such that the model optimally represents both the ALSFRS‐R and survival data.^30^, ^31^ Due to this joint optimization process, the timing of death, or the reason for dropout, lead to different patient trajectories and may prevent bias when used appropriately.^12^, ^29^

## A SIMULATED COMPARISON

By means of simulation, we will illustrate the impact of each of the discussed methods in a hypothetical clinical trial setting. In this setting, treatment has no effect on the ALSFRS‐R but produces harmful side‐effects, doubling the hazard for death in the treatment arm. This results in a 12‐month survival probability of 85% for patients allocated to placebo, and 75% for patients allocated to treatment. Suppose we enroll 100 patients per arm, where ALSFRS‐R is collected at months 0, 1, 2, 4, 6, 8, 10, and 12. Furthermore, let us assume that, based on previous literature,^13^ patients decline on average by 1.06 ALSFRS‐R points per month, where each point loss in ALSFRS‐R increases the immediate risk of death by 13%. Based on these settings, the change from baseline is, on average, 12.72 ALSFRS‐R points for both treatment arms. **Table** **2** provides the estimated mean per treatment arm, together with the treatment effect, for each method. For the sake of illustration, we made the while‐alive estimand comparable to the hypothetical estimand by multiplying the estimated monthly progression rate by 12.

**Table 2:** Estimated treatment effect on functional decline for a hypothetical randomized controlled trial with fatal side effects

Estimand	Mean placebo (at month 12)	Mean treatment (at month 12)	Mean difference (treated minus placebo)	One‐sided rejection proportion	
				Difference ≤ 0	Difference ≥ 0
**Truth** (generated via joint model)	−12.72	−12.72	0.000	0.0250	0.0250
Hypothetical estimand					
Survivor analysis (complete case)	−11.47	−10.64	0.838	0.0058	0.0771
Last observation carried forward	−11.48	−10.58	0.900	0.0037	0.1028
Worst score imputation	−15.01	−16.82	−1.813	0.1328	0.0024
Mixed model for repeated measures	−11.56	−11.45	0.104	0.0193	0.0291
While‐alive estimand					
Regression model (two‐stage)	−12.85	−12.92	−0.070	0.0235	0.0256
Random‐slopes model (one‐stage)	−12.66	−12.59	0.067	0.0219	0.0290

The while‐alive estimand targeted the progression rate per month; results were multiplied by 12 to make them comparable. Results are based on 100,000 iterations; the joint modeling framework was used to simulate conditional ALSFRS‐R and survival data; treatment resulted in a hazard ratio of 2.0; simulation parameters are described elsewhere (100 patients per arm).^13^

ALSFRS‐R, Amyotrophic Lateral Sclerosis Functional Rating Scale–Revised.

For the hypothetical estimand, the survivor analysis, last observation carried forward, and worst case imputation led to considerable deviating estimates, falsely suggesting a beneficial or harmful effect on ALSFRS‐R. The bias was reduced when using the MMRM, but underestimation of the mean change from baseline remains. To put the observed differences into context: a 2.5‐point difference after 24 weeks was considered to be sufficient to market a therapy for ALS in several countries.^32^ The one‐stage and two‐stage approaches resulted, in this simulation setting, in a closer approximation to the truth, though results may change when simulating different missing data mechanisms or nonlinear functional trajectories.^33^, ^34^ Interestingly, the MMRM and random‐slopes model rejected the null hypothesis of no effect slightly more often in favor of the treatment even though treatment has no effect on function and shortens survival time. Though the differences are small, these findings are comparable to a previous study^12^ and demonstrate the potential problems when our assumptions are not entirely accurate.

## THE COMPOSITE STRATEGY

As an alternative to the while‐alive and hypothetical strategy, we can integrate death with the variable of interest and make death part of the primary end point definition. Consequently, the composite analytical strategy does not require data after death.^35^ An important objective is to define a composite with the ALSFRS‐R in which the occurrence of death does not result in a “good” outcome.^24^, ^25^ A simple method, for example, is to score deceased patients worse than patients who survive prior to ranking their ALSFRS‐R outcomes. As a consequence, however, the treatment effect that we estimate in a composite estimand no longer solely reflects the improvement in function but also contains the improvement in survival.

In ALS clinical trials, two common composite strategies include (i) giving all deceased patients the worst theoretical score on ALSFRS‐R (0), or (ii) ranking patients based on their time of death and ALSFRS‐R outcome. The first strategy scores all deceased patients equally and does not distinguish when the patient has died during the study. Rank‐based methods, such as the combined assessment of function and survival (CAFS),^11^ or variants thereof,^36^ improve on this by giving the first patient who dies the lowest score, and the patient who survives with the best ALSFRS‐R outcomes the highest score. This reduces the number of “tied” observations and increases statistical power.

Nevertheless, a disadvantage of rank‐based methods, and composite strategies in general, is that the interpretation and quantification of the treatment effect is less straightforward.^35^ For a rank test, we could express the effect size as a winning probability,^37^ reflecting the probability that a random patient in the treated arm has a better outcome than a random patient in the placebo arm. This probability, however, may be difficult to grasp for a patient, especially since the effect could be driven by either an improvement in function or survival, and it does not provide an exact quantity that is relatable to daily life. The time to a composite event end point, such as the time to a 6‐point decrease in ALSFRS‐R or death,^38^ could be considered as an alternative, and may be easier to interpret, as it reflects the probability of an unfavorable event in the following X months, regardless of survival.^24^ A composite event, however, considers each component to be of equal importance, which may not always be appropriate when the clinical impact of components differs significantly.

Importantly, most of the composite methods use only part of the data: Patient outcomes are based on either their survival time *or* their ALSFRS‐R score. This may not only lead to a suboptimal use of information, but could also “dilute” treatment effects if the components of the composite are contributing disproportionally. For example, if 20% of the patients die at the end of the trial, 80% of the ranking scores are based on ALSFRS‐R data. If treatment selectively improves survival and not the ALSFRS‐R, the treatment effect that we observe on the composite end point can only be driven by 20% of the patients, while the remaining 80% of the patients provide no meaningful information for the effect estimate. Multivariate methods, such as a joint model that incorporates all available information for ALSFRS‐R and survival, may provide a more powerful alternative,^13^ but this may be at the price of increasing the risk of defining an erroneous model.^39^

## TO COMBINE, OR NOT TO COMBINE?

Overall, each of the three strategies to address death have strengths and weaknesses, irrespective of which method is chosen (**Table** **3**). For clinical trials with a longer follow‐up duration, where death rates are expected to exceed 20–30%,^10^ the hypothetical estimand may be the least informative for patients and physicians as it reflects an imaginary world applicable to only a few patients. Nevertheless, the hypothetical estimand holds value for shorter studies with a negligible number of deaths as it may require fewer assumptions about the treatment effect than the while‐alive estimand, and its interpretation is more straightforward than for a composite. The primary choice for longer, pivotal clinical trials in ALS seems, however, to be composed of either the while‐alive strategy or a strategy that combines the ALSFRS‐R and mortality into a composite end point.

**Table 3:** Summary of the hypothetical, while‐alive, and composite estimand for ALS clinical trials

	Hypothetical strategy	While‐alive strategy	Composite strategy
Example research question	In patients with ALS who survive 12 months, what is the between‐group difference in mean daily functioning, as measured by the ALSFRS‐R total score, between treatment and placebo, at 12 months after randomization?	In patients with ALS, what is the between‐group difference in mean rate of functional loss, as measured by the ALSFRS‐R total score, between treatment and placebo, over 12 months after randomization or until death (whichever occurs first)?	In patients with ALS, what is the probability that a random patient on treatment has a longer survival or better daily functioning, as measured by the ALSFRS‐R total score, compared with a random patient on placebo, at 12 months of treatment?
Benefits	Clinically relevant and easy to explain to patient if survival probability is high Powerful strategy when death rates are low	Clinically relevant and easy to explain to patient, irrespective of survival probability	Depending on method, fewer assumptions and no need to extrapolate after death Estimates the totality of the treatment effect on both ALSFRS‐R and survival
Disadvantages	Assumptions are needed about the patient‐specific trajectory before and after death, which can introduce bias in effect estimate Interpretation of and applicability to the intended population becomes complicated when death rates are high	Assumptions are required about the patient‐specific trajectory over time Assumptions are required about the response to treatment over time	Interpretation of the treatment effect is less straightforward and can be driven by each component Loss of information and/or power when death rates are low, or treatment affects only one component

ALS, amyotrophic lateral sclerosis; ALSFRS‐R, Amyotrophic Lateral Sclerosis Functional Rating Scale–Revised.

An argument in favor of the composite strategy is that it reflects the totality of the treatment effect. Especially in a disease such as ALS, for which there is an urgent and enormous unmet medical need, one may prioritize simple “yes/no” decisions about the success of the new treatment over the exact interpretation of its benefits.^15^ Suppose a treatment reduces the ALSFRS‐R progression rate but at the same time affects an important prognostic mechanism that is not captured by the ALSFRS‐R (e.g., weight loss or cognitive decline). Solely measuring the ALSFRS‐R would only partially capture the treatment response and may falsely discard an efficacious drug. In these settings, capturing the totality of the treatment effect on a composite of ALSFRS‐R and survival holds clear advantages over the while‐alive strategy.

The success of the composite strategy, however, depends on how well one is able to capture the treatment effects on the individual components. If one of the components is nearly unquantifiable, the effect observed on the composite end point can only be driven by other components. The same holds true for the ALSFRS‐R and survival: In a 6‐month study on 100 patients, the expected number of deaths is 0 to 5.^10^ As such, any treatment effect on the composite is virtually solely driven by the ALSFRS‐R. Under these circumstances, even if treatment improves additional prognostic mechanisms, the disadvantages of using a composite may not outweigh the benefits of other strategies.

**Figure 2:**
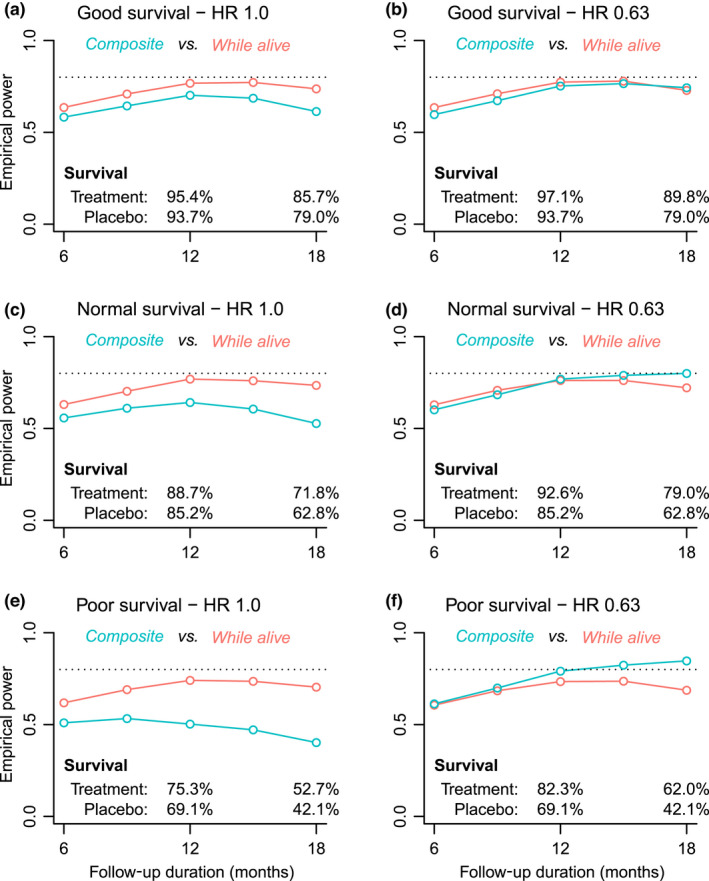
Empirical power of the while‐alive and composite strategies. Data were simulated for a hypothetical randomized controlled clinical trial where treatment reduces ALSFRS‐R progression rate by 30% (**a, c, and e**). In scenarios (**b, d, and f**), an additional treatment effect on survival, independent of the ALSFRS‐R, was added with an HR of 0.63.^40^ The composite strategy was based on the CAFS (combined assessment of function and survival) end point;^11^ the while‐alive strategy was approached using a random‐slopes model. Each scenario was simulated 10,000 times; exact simulation details are provided elsewhere.^13^ HR, hazard ratio.

Thus, the choice to combine the ALSFRS‐R and survival is a trade‐off between the disadvantages and advantages of each strategy. This is for an important part driven by the number of deaths and depends, therefore, on the underlying survival distribution and the duration of the study.^10^ This trade‐off is illustrated in **Figure** **2** for a while‐alive strategy using a random‐slopes model, and a composite strategy using the CAFS in six hypothetical scenarios with different survival rates. In each scenario, treatment reduces the ALSFRS‐R progression rate by 30%. In scenarios **b**, **d**, and **f**, treatment additionally affects a prognostic mechanism not captured by the ALSFRS‐R. As can be seen, even though the CAFS captures the totality of the treatment effect, this only leads to improved statistical power over the while‐alive strategy in longer trials, or when death rates are high. On the other hand, if there is no additional prognostic benefit of treatment, or survival rates are low, the CAFS underperforms due to the loss of information.^11^, ^12^, ^13^ As such, the decision to use a composite strategy, especially when the primary goal is to find an efficacious drug, should be carefully weighed against the hypothesized treatment benefit, intended duration of the trial, and the targeted patient population.

## FINAL REMARKS

In this paper, we have provided a general overview of the considerations that play a role when addressing death in efficacy end points for ALS clinical trials. The key consideration is that the chosen strategy has a major impact on the research question being answered. Not every analytical strategy will necessarily provide a clinically relevant answer; its relevance changes as a function of the intended trial duration, hypothesized treatment effect, and included population. Therefore, clarifying at the design stage exactly what the research question is that one aims to answer will guide the discussion about which analytical strategy for handling death is required and will better direct the planning, design, and analysis of the study.

It is important to note that we have only discussed the strategy for addressing death. The estimand for other intercurrent events, e.g., treatment discontinuation due to adverse events or nonadherence, require different strategies. In the end, the final estimand of a trial will be composed of different strategies to address each intercurrent event individually.^22^, ^25^ Moreover, in this paper we have discussed methods commonly used to address death in ALS clinical trials; we did not aim to provide an exhaustive list of all possible options, or provide an in‐depth review of each method. Future work can play a major role in this regard. Innovative methods may overcome the limitations of current analytical strategies and allow further scrutiny of when a particular strategy might be most beneficial. The simulations provided in this paper are intended simply as an illustration, and the trade‐off between strategies may change when alternating the expected treatment response, the relationship between function and mortality, and using different design settings.

## FUNDING

R.P.A.v.E. is supported by the Dutch Research Council (Rubicon, 452019301); Y.L. is partially supported by the National Institutes of Health Research (5UL1TR003142‐03, 5R01HL08977810, 3P30CA12443512).

## CONFLICT OF INTEREST

The authors declared no competing interests for this work.
